# Does Reduced Ball Inflation Pressure in Association Football Decrease Head Impact Kinematics?

**DOI:** 10.1007/s10439-025-03804-0

**Published:** 2025-08-04

**Authors:** Rory England, Suzie Liverseidge, Yusuke Miyazaki, Samuel W. Oxford, Ieuan Phillips, Jon Farmer

**Affiliations:** 1 Wolfson School of Mechanical, Electrical and Manufacturing Engineering, Loughborough University, Loughborough, LE11 3TU UK; 2 Department of Systems and Control Engineering, Institute of Science Tokyo, Tokyo, Japan; 3 Centre for Physical Activity Sport and Exercise Science, Coventry University, Priory Street, Coventry, CV15FB UK

**Keywords:** Head injury, Brain injury, Injury biomechanics, Football, Soccer, Degenerative brain health, Heading, Head impacts

## Abstract

**Purpose:**

Apparent degeneration in brain health due to heading the football is amongst the most pressing and contentious health-related questions in sport (Keogh F, Pirks N (2024) “Pain was sickening”—Ex-players on heading fears. In: BBC). The purpose of this study was to thoroughly explore the effectiveness of reduced inflation pressure as an intervention to reduce head kinematics from a ball to head impact. The influence of impact location, head orientation, neck flexion angle and ball type on the intervention were experimentally investigated.

**Method:**

A Hybrid III head and neck was impacted in frontal and oblique locations with two modern footballs that were projected using a bespoke launch device. Peak linear acceleration, peak angular velocity, peak angular acceleration and DAMAGE metrics were calculated for a total of 34 permutations of impact variables at two inflation pressures.

**Results:**

Magnitude was decreased (82%) or unchanged (8%) for 90% of impacts with average magnitude changes between − 2 and − 12% across the four metrics. Findings indicated that decreased inflation pressure was a positive intervention towards decreasing kinematic magnitudes in most cases. This was especially true for linear acceleration, angular velocity and angular acceleration, where 100, 97 and 100% of impacts were reduced or unchanged, respectively.

**Conclusions:**

Reduced inflation pressure was overall an effective mitigation to reduce the kinematic magnitude of heading in football based upon these four kinematic metrics despite 10% of impacts exhibiting an increase in kinematic magnitude. The DAMAGE predictor of MPS exhibited 12 out of 13 cases where magnitude increased demonstrating the capacity for decreased inflation pressure to result in increased kinematics for an angular response derived metric, indicating that reducing inflation pressure is not a universal solution. Nonetheless DAMAGE still saw a net decrease in magnitude across all impacts. Metric magnitude was found to be sensitive to head orientation, impact location and ball type, demonstrating the importance of the sensitivity analysis in this study. Two impacts were recommended to represent the worst-case ball to head impact, one in each nominal orientation. These locations contrasted with those commonly used in literature, a finding pertinent to future experimental design in football heading research.

**Supplementary Information:**

The online version contains supplementary material available at 10.1007/s10439-025-03804-0.

## Introduction

Association football (soccer) is amongst the most popular sports in the world being played by over 200 million people globally with more than 130 thousand professional players [2]. Currently there is significant research interest in the severity of the ball to head impact and the potential link to long-term degeneration of brain health in retired professional footballers [3–6]. As well as chronic neurodegenerative pathology there is also evidence of several acutely symptomatic ball to head impacts that cannot be overlooked [7, 8]. Symptoms vary widely but have included feeling dazed, tingling, dizziness, headaches, sleep issues and loss of consciousness [1, 9, 10]. Studies have measured head kinematics in vivo by instrumenting the players (e.g., instrumented mouthguards). A meta-analysis of 26 studies reported head kinematics of typical ball to head impacts as 10 to 40 g for peak linear acceleration (PLA) and 1.0 to 8.0 krad/s^2^ for peak angular acceleration (PAA) [10]. Results for PLA with custom instrument mouthguards [11–15] were on average 56% lower than those with the instrumentation attached to the surface of the head showing that instrumentation method was a significant factor for in vivo studies. The findings Barnes-Wood et al. [16] concur with this meta-analysis, reporting linear acceleration of 31.9 g, angular acceleration of 2.1 krad/s^2^ and angular velocity of 9.0 rad/s for in vivo heading of a ball (16 m/s) using an Opro (Hertfordshire, UK) instrumented gum shield mounted to the upper dentition. These are significantly below the threshold where acute symptoms might be expected [17–25]; nevertheless, given the suggested epidemiological link between repetitive head impacts and neurodegeneration, measurement and understanding of non-acutely symptomatic heading duress remains of significant interest. Data captured from human volunteers and instrumented surrogates have also been used to drive finite element modelling (FEM). The most common metric taken from FEM is maximum principal strain (MPS) which cannot be directly measured in vivo. Barnes-Wood et al. [16] reported MPS ranging from 0.07 to 0.16 (mm/mm) for 16 m/s impacts and a mean of 0.12 (mm/mm). Huber et al. [26] found that MPS was at most 0.07 (mm/mm) and quoted a 50% chance of symptomatic outcome at 0.12 (mm/mm) and a 95% chance at 0.23 (mm/mm). However, their kinematic inputs (12.3 − 17.7 g and 1.1 − 1.4 krad/s^2^) were at the lowest end of the range for PLA and PAA found in the meta-analysis by Basinas et al. [10].

Various mitigation strategies have been suggested to lessen the severity of ball to head impacts. These include using an age-appropriate ball in youth football (size and mass), strengthening athletes’ neck muscles, reducing the frequency of headers (especially those at high velocity), reducing inflation pressure of the ball, and various products marketed as football head personal protective equipment [27–30]. In 2020, the Union of European Football Associations (UEFA) issued guidance with the intention of reducing the impact severity of headers in youth football. This guidance included the recommendation to reduce the pressure of the ball to “the lowest pressure authorised by the laws of the game” [27]. The effectiveness of this intervention when considering impact location, neck orientation and ball type is not well established, especially considering the effect of an increased contact patch of the softer ball on rotational kinematics. Therefore, the aim of this study was to investigate the influence of reduced ball inflation pressure during ball to head collisions, in a range of realistic heading configurations, on the resulting head kinematics and associated injury risk metrics. The experimental research involves projecting footballs at an instrumented anthropomorphic test device (ATD) head and explores the influence of the aforementioned confounding variables.

## Materials and Methods

### Ball Selection and Mechanical Characterisation

Two commercially available balls were chosen. The Adidas Uniforia Pro (UEFA Euro 2020 match ball) was chosen as the primary ball that was used for most impacts. The Adidas Al Rihla Pro (FIFA World Cup 2022 match ball) (AR) was used as the secondary ball to investigate sensitivity to ball type. Match balls were chosen because they are the only ball type designed to be inflated up to the maximum inflation pressure permitted by FIFA regulations and because match balls have the tightest manufacturer tolerances, reducing the need for repeat testing with multiple balls. The Uniforia is a typical professional ball from the period 2012-2023 with thermally bonded laminated foam panels, latex impregnated polyester carcass, counter weighted rubber bladder, depressed seams and micro textured polymer film surface. The AR was included because it was most atypical of this period. It has macro texture surface debossing [31] and has been reported by players to have a stiffer feeling shell [32].

The balls were mechanically characterised for mass, moment of inertia (MOI), coefficient of restitution (COR), quasi-static compressive stiffness and diameter. Each ball was characterised at 0.6 bar and 1.0 bar inflation pressures and were chosen as the upper limit of the manufacturer inflation guidance and the lower limit of the FIFA regulations on inflation pressure (0.6 to 1.0 bar). Characterisation was repeated three times for the valve axis and an axis perpendicular to this (Table 1).

**Table 1:** Definition of test methods used to characterise the footballs

Test	Description
Mass	Measured using Mattler Toledo SB8001 scales (Columbus, US). [Uncertainty ± 0.5 g]
Diameter	Measured using an RS Pro Vernier Height Guage (Corby, UK ) in two locations and repeated five times across the valve and an orthogonal axis independent of the valve. [Uncertainty ± 0.01 mm]
Moment of inertia	Measured using Inertia Dynamics LLC inertia measurement device (New Hartford, US). The measurement was repeated five times and about two axes. Across the valve and an orthogonal axis independent of the valve. [Uncertainty ± 0.5 %]
Coefficient of restitution	Measured following the FIFA quality programme methodology [33]. Repeated five times with three impact locations. On the valve, opposite the valve and an orthogonal axis independent of the valve.
Compressive stiffness	The ball was compressed between two 150 mm diameter compression plates by 50 mm using an Instron 5569 universal mechanical test machine (Norwood, US) at a rate of 5 mm/s. A 10 kN load cell was used to measure the force as the ball was dynamically compressed [uncertainty ± 0.25%]. Repeated 5 times in two locations. Across the valve and an orthogonal axis independent of the valve. Stiffness was calculated at maximum displacement because the force-displacement curves showed that the force progression was relatively linear over the 50 mm displacement.

### Experimental Ball to Head Impact Setup

Laboratory representations of ball to head collisions were established by projecting a ball with a bespoke push-action pneumatic cannon with near zero spin. A nominal ball impact speed of 17 m/s was specified to represent a ball to head collision after a corner kick [34]. The cannon was calibrated for speed and impact location resulting in a standard deviation of ± 0.17 m/s and ± 2 mm, respectively. Speed was measured using laser gates less than 1 m prior to impact and a sample of measurements were verified against high speed video. Each impact was captured with high-speed video at 5400 fps using a Photron Fastcam SA1.1 camera (Tokyo, JP) and PIXAPRO LECO500 lights (Brierley Hill, UK). Each impact condition was repeated three times.

**Fig. 1:**
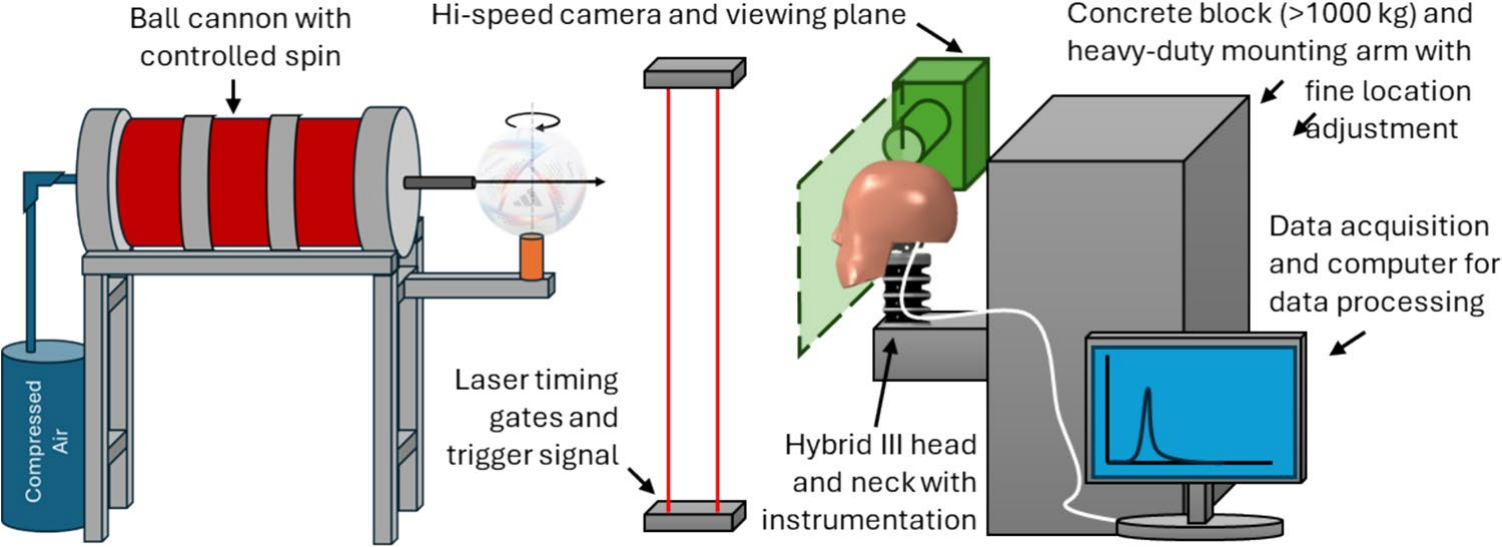
Annotated diagram of experimental setup

The Hybrid III head and neck were chosen for their wide availability, durability and previous use in this and similar contexts [35]. The headform was instrumented with a DTS 6DXPro (Calabasas, US) triaxial angular rate sensor (±18,000 deg/s) and triaxial accelerometer (±2000 g) mounted at the headform’s centre of mass. The base of the neck was rigidly mounted to a heavy-duty metal frame with fine location adjustment, which in turn was mounted to a concrete block. The experimental setup is shown in Fig. 1. During testing, the headform was inspected at regular intervals and where necessary wiped with a dry cloth to remove any residual material deposits from the ball which built up over time to avoid ball-head interaction changes between trials.

**Fig. 2:**
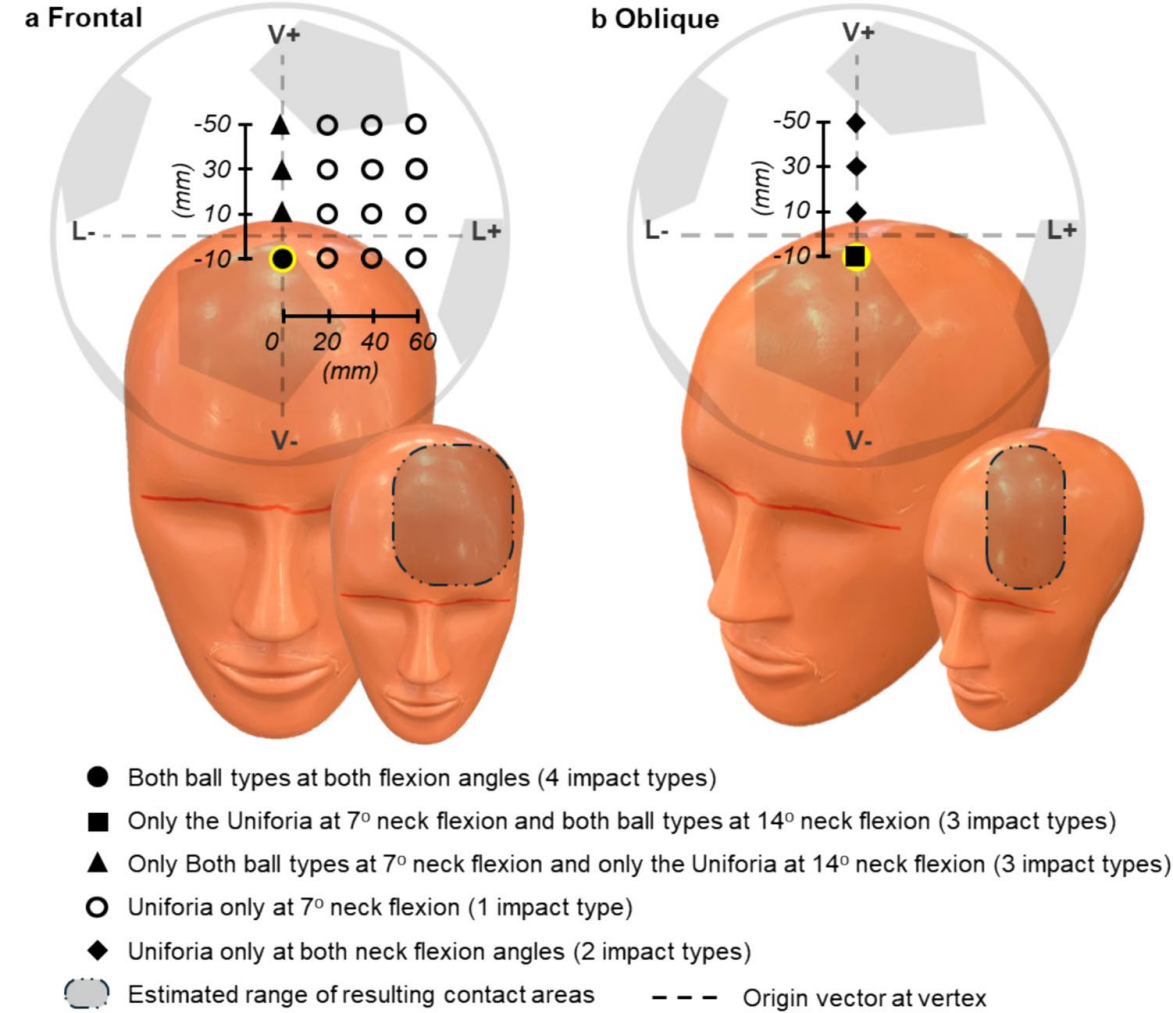
Visual depiction of tested impact locations (approximate relative scale). Velocity vector into the page. The resulting contact location was in the lower third of the ball due to geometric interaction

In previous experimental research of football heading, impact location has been described as frontal, lateral or oblique [16, 26] with limited quantification of these locations to allow others to recreate them. In this study, two primary impact locations were defined anatomically as the ‘neutral-frontal impact’ and the ‘neutral-oblique impact’. These were representations of two distinct heading techniques, a directed header and a glancing header. The frontal orientation was defined with the midsagittal plane of the headform aligned to the ball’s velocity vector (Fig. 2.a). In each of these orientations a target coordinate system was defined by aligning the velocity vector of the centre of the ball with the Vertex of the head. This was the origin from which each impact location was defined by a vertical and lateral translation. For the two primary impact locations the ball target vector was translated 10 mm inferior of the Vertex. This was the most inferior impact location possible on the forehead without encroaching on the eye sockets. The neck flexion angle for these primary impacts was defined as + 7° relative to the mounting bracket of the Hybrid III neck which places the Frankfort plane parallel to the ground. This was informed by reprocessed kinematic data which showed that average neck flexion angle was in the range of + 7° to + 14^o^ [36] (Table 2).

**Table 2:** Definition of primary impact locations

Primary impact locations	Axial rotation	Neck flexion	Target location (vector relative to vertex)		Ball
			Vertical	Lateral	
Neutral-Frontal	0^o^	7^0^	− 10 mm	0 mm	Uniforia
Neutral-Oblique	45^o^	7^o^	− 10 mm	0 mm	Uniforia

In the absence of a well-defined impact location in the literature, a sensitivity analysis was conducted in 20 mm increments to understand the influence of vertical and horizontal offsets with respect to the primary impact locations (Fig. 2). For the frontal impact this was investigated in the vertical and lateral directions (up to 60 mm offsets) and for the oblique impact this was investigated only in the vertical (up to 60 mm offset). Sensitivity to neck flexion angle was investigated with a selection of + 14° flexion cases. In total 68 combinations of ball type, neck flexion angle, impact location and inflation pressure were investigated with three repeats for each condition.

### Data Processing, Analysis and Statistics

Kinematic data was captured at 100 kHz and processed using MATLAB (Natick, US) with a 1650 Hz low pass filter for the linear acceleration data, a 1650 Hz low pass filter for the angular velocity data and a 300 Hz low pass filter for angular velocity data before differentiation to angular acceleration. Peak values of linear acceleration (PLA), angular velocity (PAV) and angular acceleration (PAA) were extracted. The metric DAMAGE was also calculated using MATLAB as a predictor of maximum principal strain of the brain [37] which was correlated to results from computational modelling (Equation 3). These four metrics have been previously correlated with impact severity [18–25, 38–42]. Conservative symptomatic thresholds (CST) were established from literature (Table 3) to enabled comparison between the magnitude of different metrics that correlated with the onset of acute symptoms. Thresholds for mild traumatic brain injury (mTBI) were prioritised above thresholds for more severe forms of brain injury. Equation 1 (mean minus half a standard deviation) was used to qualify these as conservative thresholds. Results were compared using descriptive statistics including the mean and an independent t-test (Equation 2) with p-value of 0.01.

**Table 3:** Table establishing CST for expected symptomatic outcome based upon the four metrics used in this study and the references used to support this threshold

Metric	CST	References
PLA	51 g	[18–22]
PAV	22.7 rad/s	[18, 23].
PAA	6.6 krad/s^2^	[18, 20, 21, 24, 25, 42]
DAMAGE (MPS)	0.15 MPS	[38–41]

1 $$CST= \overline{X }-\left(0.5 \times \sigma \right)$$

Equation 1 – Equation for CST.

2 $$t= \frac{\overline{{x }_{1}}+\overline{{x }_{2}}}{\sqrt{\frac{{{s}_{1}}^{2}}{{n}_{1}}+\frac{{{s}_{2}}^{2}}{{n}_{2}}}}$$

Equation 2 – Equation for independent t-test.

3 $$DAMAGE = \beta \max_{t} \left\{ {\left| {\mathop{\delta }\limits^{\rightharpoonup} \left( t \right)} \right|} \right\}$$

Equation 3 –DAMAGE metric [37] where $\mathop{\delta }\limits^{\rightharpoonup}$ is angular displacement with time.

## Results

### Ball Characterisation

The two ball types had small differences (~1%) between their measured mass and diameter. When inflation pressure was increased, both balls increased in mass and diameter by 0.002 Kg and 0.5 mm, respectively. Measured stiffness was higher in the valve axis for both balls (+4.1 to + 6.3%) but was affected to a greater extent by inflation pressure. The Uniforia ball increased by 38 and 35% for the independent and valve axes, respectively. The AR ball increased by 28 and 30% for the same axes, respectively. COR was consistently greatest opposite the valve, followed by the independent axis and lowest on the valve. The increase in COR due to increased inflation pressure (averaged across the three test axes) was 0.033 (AR) and 0.030 (Uniforia), equating to around 4% (Table 4).

**Table 4:** Results of ball characterisation tests presented as mean (standard deviation)

		Uniforia		Al Rihla	
	Test axis	0.6 bar	1.0 bar	0.6 bar	1.0 bar
Mass (Kg)		0.435	0.437	0.431	0.433
Diameter (mm)	Valve	217.0 (0.06)	217.6 (0.13)	216.0 (0.12)	216.4 (0.03)
	Independent	216.7 (0.18)	217.5 (0.04)	218.2 (0.01)	218.3 (0.40)
MOI (Kg.cm^2^)	Valve	32.04 (0.00)	32.34 (0.10)	31.57 (0.00)	31.74 (0.00)
	Independent	32.87 (0.14)	33.18 (0.01)	32.53 (0.01)	32.80 (0.00)
Stiffness (N/mm)	Valve	28.8 (0.49)	38.9 (0.49)	29.9 (0.22)	38.3 (0.03)
	Independent	27.0 (0.29)	37.3 (0.11)	28.0 (0.54)	36.3 (0.52)
COR	On valve	0.781 (0.01)	0.818 (0.00)	0.776 (0.00)	0.811 (0.00)
	Opposite Valve	0.800 (0.01)	0.825 (0.00)	0.794 (0.00)	0.823 (0.00)
	Independent	0.791 (0.00)	0.818 (0.01)	0.783 (0.00)	0.818 (0.01)

### Head Kinematics of Primary Impacts

The results for the primary impact locations, neutral-frontal and neutral-oblique at the 0.6 and 1.0 bar inflation pressures for the primary ball type (Uniforia) (Table 6) are indicative of highly repeatable experimental methods as the standard deviations values do not exceed 2.1% of their associated magnitude for all results. Reduced ball inflation pressure decreased the magnitude of all metrics in both impact locations. The reduction in PLA and PAA were largest (− 14 to − 21%) whereas the reduction in PAV and DAMAGE was all smaller than − 10%. The reduced inflation pressure had more of an effect in the frontal impact location for three out of the four metrics (PLA, PAA and DAMAGE). At 0.6 bar the oblique impact resulted in greater magnitudes (+5 to + 19%) for every metric compared to the frontal location. PAV and PAA saw the largest increase (19%) relative to the frontal impact. DAMAGE and PLA showed increases of 9% and 5%, respectively. Similarly, at the 1.0 bar ball pressure, the predicted DAMAGE showed an increase of 6% at the oblique impact location compared to the frontal impact. Further, PAV and PAA magnitudes were 26% and 19% greater, respectively, agreeing well with the results at 0.6 bar. In contrast, the magnitude of PLA at 1.0 bar decreased at the oblique impact location by 3% compared to the frontal location (Table 5).

**Table 5:** Key metrics for the two primary impact locations (neutral-frontal and neutral-oblique) at the two pressures with the primary ball (* statistically significant p = 0.01), (mean of three impacts with standard deviation in brackets)

Impact #	Ball	Pressure (Bar)	Orientation	Neck Flexion)	Vertical (mm)	Horizontal (mm)	Repeats	PLA (g)		PAV (rad/s)		PAA (rad/s^2^)		DAMAGE (mm/mm)	
#1	Uniforia	1.0	Frontal	7 ^o^	− 10	0	3	34.28	(0.13)	10.74	(0.05)	3.90	(0.02)	0.105	(0.000)
#2	Uniforia	0.6	Frontal	7 ^o^	− 10	0	3	27.18	(0.06)	10.47	(0.04)	3.14	(0.04)	0.096	(0.001)
#3	Uniforia	1.0	Oblique	7 ^o^	− 10	0	3	33.23	(0.56)	13.41	(0.21)	4.63	(0.10)	0.111	(0.002)
#4	Uniforia	0.6	Oblique	7 ^o^	− 10	0	3	28.53	(0.09)	12.42	(0.04)	3.73	(0.06)	0.104	(0.001)

T-test		Subject of T-test		Percentage change in magnitude											
(#1 v #2)		0.6 and 1.0 bar at Frontal		− 21%*			− 3%*			− 20%*			− 8%*		
(#3 v #4)		0.6 and 1.0 bar at Oblique		− 14%*			− 7%*			− 19%*			− 6%*		
(#2 v #4)		Oblique and Frontal at 1.0 bar		− 3%*			25%*			19%*			6%*		
(#1 v #3)		Oblique and Frontal at 0.6 bar		5%*			19%*			19%*			9%*		

### Impact Configuration Sensitivity Analysis

**Fig. 3:**
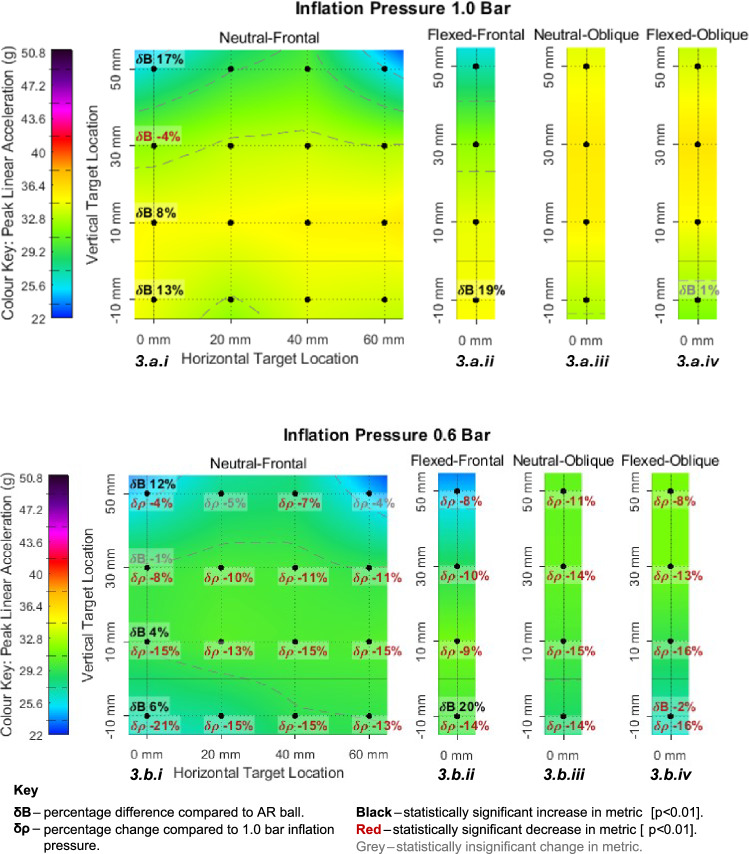
PLA heat maps with percentage difference in PLA due to decreased inflation pressure (lower number) and alternative ball model (upper number)

**Fig. 4:**
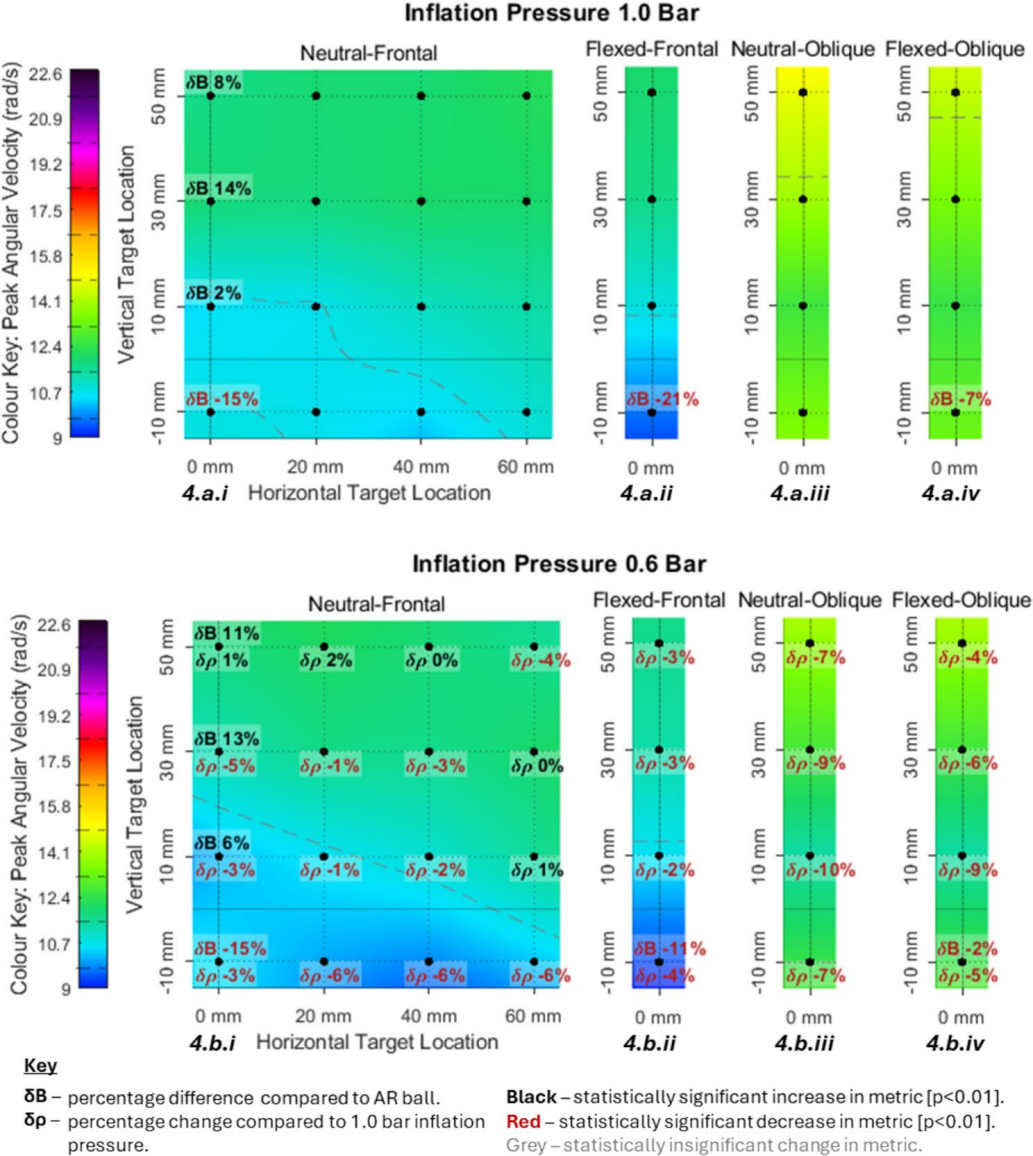
PAV heat maps with percentage difference in PAV due to decreased inflation pressure (lower number) and alternative ball model (upper number)

**Fig. 5:**
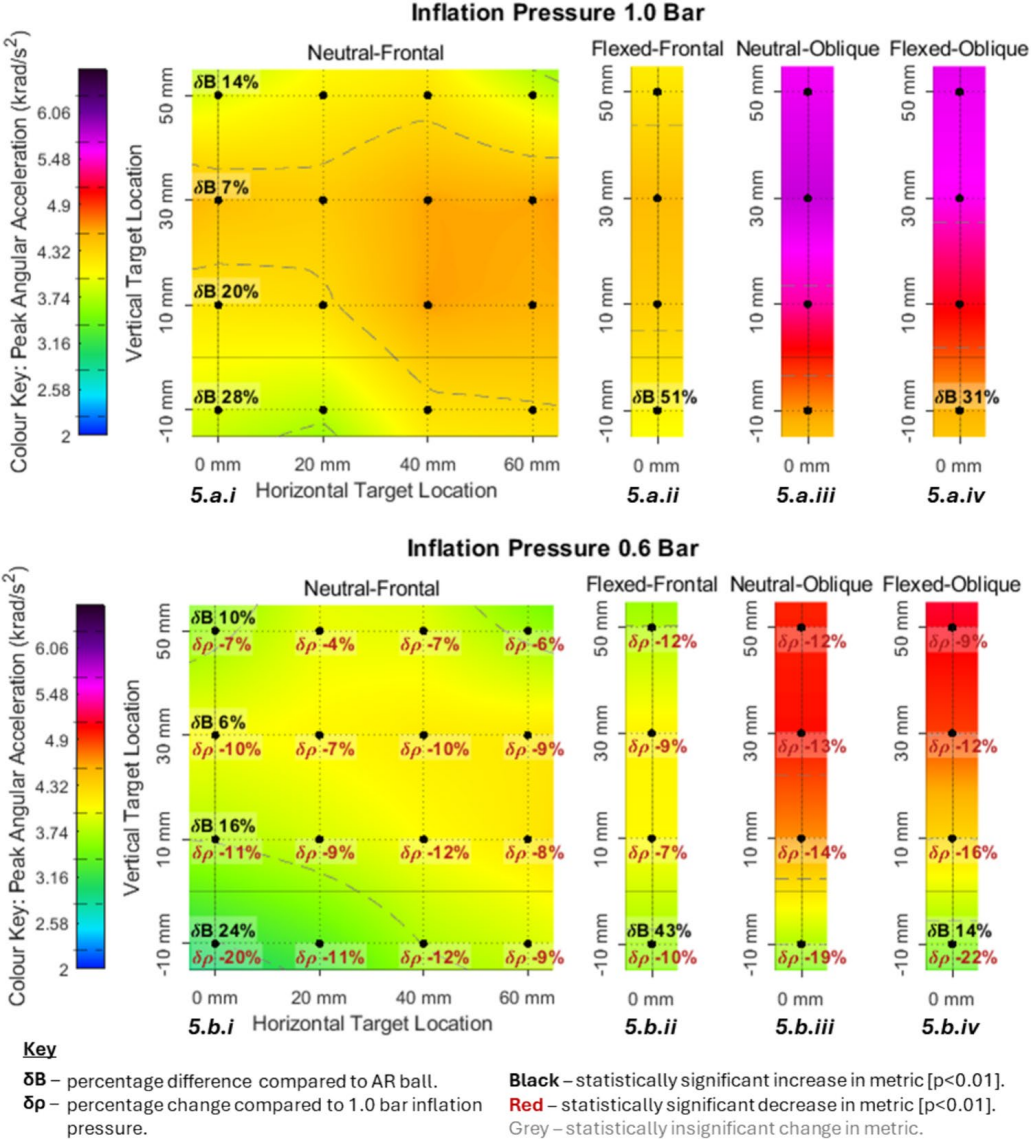
PAA heat maps with percentage difference in PAA due to decreased inflation pressure (lower number) and alternative ball model (upper number)

**Fig. 6:**
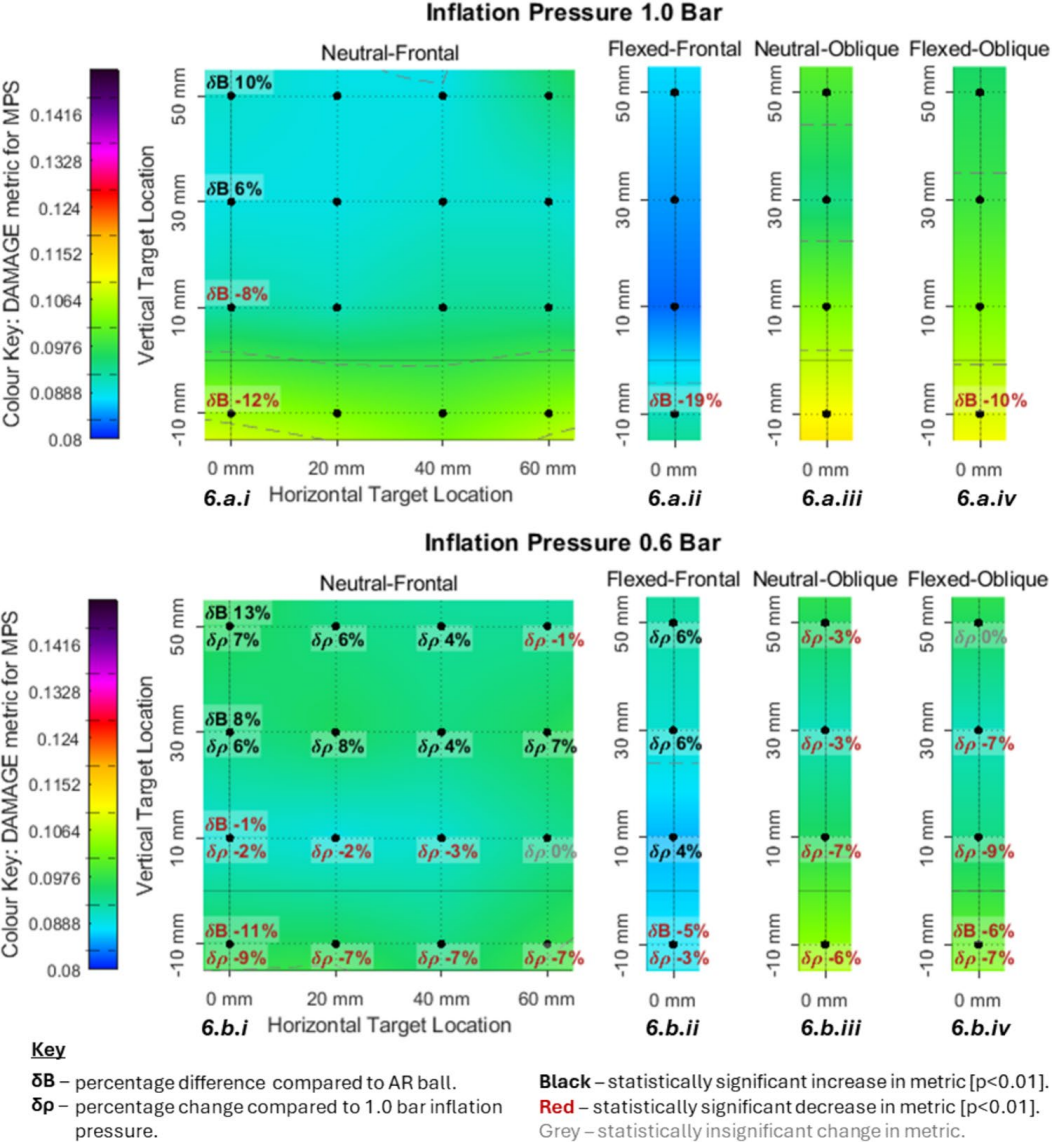
DAMAGE metric heat maps with percentage difference in DAMAGE due to decreased inflation pressure (lower number) and alternative ball model (upper number)

Figures 3, 4, 5 and 6 visually present the magnitude (average of three repeats) of four kinematic impact metrics across 68 impact conditions. These include the two primary impacts (neutral-frontal and neutral-oblique) but quantify the sensitivity of changing impact location, neck flexion angle and ball type. Figures 3, 4, 5 and 6 each present eight separate colour maps. Colour maps a.i to a.iv (across the top) present the results at 1.0 bar condition and colour maps b.i to b.iv (across the bottom) present the corresponding results at 0.6 bar condition. The pair of colour maps a.i and b.i present the results for the vertical and horizontal sensitivity grid (4x4), starting from the primary neutral-frontal impact. The pair of colour maps a.ii and b.ii present a vertical sensitivity analysis for the flexed-frontal impact which was equivalent to the first column of the neutral-frontal impacts (a.i and b.i) but with 14° of neck flexion. The colour maps a.iii and b.iii present the vertical sensitivity analysis starting from the primary neutral-oblique impact location. The colour maps a.iv and b.iv present a vertical sensitivity analysis for the flexed-oblique impact which was equivalent to the neutral-oblique impacts but with 14° of neck flexion.

The numbers on Figs. 3, 4, 5 and 6 show the percentage magnitude difference of the plotted value compared to the equivalent impact at lower inflation pressure and the percentage change compared to the secondary ball (AR). A positive number denotes that the value labelled was larger than the value it is being compared against. Positive changes in magnitude are displayed in black and negative changes are displayed in red. A comparison is made with the secondary ball (AR) in six locations, this is always plotted above the labelled point and symbolically denoted ‘change due to ball’ (δB). For the tests at 0.6 bar (b.i to b.iv) a comparison is also made with the tests at 1.0 bar. This is always plotted below the labelled point and symbolically denoted ‘change due to pressure’ (δρ).

Each colour scale was set relative to the CST in Table 3 to allow comparison between the metrics. The upper colour limit was the CST value (Table 3). The lower limit was the minimum value from the data collected, which is strongly believed to be acutely asymptomatic due to the low frequency of acutely symptomatic ball to head impacts [7, 8].

#### Sensitivity Analysis for PLA

Figure 3.a.i to 3.b.iv presents all results for all conditions for PLA. PLA ranged from 24.7 to 31.4 g for the 0.6 bar state and 25.7 to 35.9 g for the 1.0 bar condition. For each ball pressure condition the maximal region was found across 10–15 discrete locations which were within 5% of the absolute maximum. Twenty-six out of twenty-eight discrete impact locations resulted in a statistically significant (*p* < 0.01) reduction in PLA due to decreased inflation pressure. The results ranged from 48 to 70% of the CST represented by the range of the colour pallet.

Across all configurations and locations, the mean reduction in PLA due to decreased inflation pressure was 11.7%. The largest decrease in PLA occurred at the more inferior impact locations (Fig. 3.b.i to 3.b.iv). The mean decreases in PLA at the heights of − 10, 10, 30 and 50 mm were − 15.4%, − 14.0%, − 11.0% and − 6.2%, respectively. The maxima of peak values for neutral-frontal impacts (Fig. 3.a.i) occurred at the height of 0 − 20 mm across all horizontal locations for the 1.0 bar condition. This maxima band translated vertically upwards to 10 − 30 mm for the 0.6 bar condition (Fig. 3.b.i). Increased neck flexion (Fig. 3.a.ii and 3.b.ii) resulted in a small change to the maximal PLA (+2.6% at 0.6 bar and − 2.1% at 1.0 bar). The maxima occurred at a lower height of − 10 − 0 mm for the 1.0 bar condition (Fig. 3.a.ii) compared to 0 − 20 mm for the 0.6 bar condition (Fig. 3.b.ii).

The mean PLA magnitude was on average 1.5% higher in the oblique conditions compared to the frontal. The highest PLA magnitude response for the neutral-oblique and flexed-oblique impacts at 1.0 bar (Fig. 3.a.iii and 3.a.iv) occurred at a height of 10 − 40 mm. For the 0.6 bar state (Fig. 3.b.iii and 3.b.iv) the maxima transformed upwards to 10 − 50 mm and this was more pronounced in the flexed-oblique condition (Fig. 3.b.iv). The lowest magnitude responses occurred at the most superior locations for the frontal impacts (Fig. 3.a.i, 3.b.i, 3.a.ii and 3.b.ii) and the most inferior location for the oblique impacts (Fig. 3.a.iii, 3.b.iii, 3.a.iv and 3.b.iv).

It is important to note that in Fig. 3 that the percentage change is displayed from the perspective of the primary ball compared to the secondary ball. The ball type had an inconsistent influence over resulting PLA. For the primary ball, PLA ranged from − 4 to + 20% compared to the secondary ball. At both pressures, the secondary ball did not change the result for the flexed-oblique cases (Fig. 3.a.iv and 3.b.iv) but caused a 19 − 20% reduction in PLA for the flexed-frontal case (Fig. 3.a.ii and 3.b.ii). In the neutral-frontal cases the secondary ball resulted in a mean decrease of 5.8% and 8.5 % for the 0.6 bar condition (Fig. 3.b.i) and 1.0 bar condition (Fig. 3.a.i), respectively. At both pressures the secondary ball resulted in a slight increase in PLA at 30 mm vertical offset but decreases at all other locations.

#### Sensitivity Analysis for PAV

PAV ranged from 9.9 to 14.8 rad/s (Figure 4.a.i to 4.b.iv.). Across all impacts reduced inflation pressure resulted in an average decrease in PAV of 3.8%. The range of values were 44–65% of the CST and are highlighted by the range of the colour pallet.

For the frontal cases PAV ranged from 9.9 to 12.2 rad/s (Fig. 4.a.i, 4.a.ii, 4.b.i and 4.b.ii.). The neutral-frontal cases had maxima across eight of the more superior impact locations for both pressure conditions (Fig. 4.a.i and b.i). These discrete points were within 5% of the overall maximum. This included the entire top row of impacts, the three most lateral impacts of the + 30 mm row and the most lateral impact of the + 10 mm row. This formed a diagonal separation between the minimum region in the inferior-medial corner. The flexed-frontal cases (Fig. 4.a.ii and b.ii) had a smaller magnitude than the neutral-frontal cases (− 7.5% at 1.0 bar and − 5.4% at 0.6 bar) with maxima at the two most inferior locations. The maxima at the more superior locations were similar magnitude to the neutral-frontal cases (− 1.9% at 1.0 bar and − 3.8% at 0.6 bar).

The oblique cases ranged from 11.4 to 14.8 rad/s (Fig. 4.a.iii, b.iii, a.iv and b.iv.) and were generally larger in magnitude than the frontal cases. The lowest magnitude PAV in the oblique cases was similar in magnitude to the maxima of the frontal cases (+3.0% at 1.0 bar and − 4.9% at 0.6 bar). The maximum PAV for the oblique cases was at the most superior location and was greater than the maximum for the corresponding frontal cases in terms of flexion and ball pressure by between 14 and 22 %. The mean decrease in PAV due to decreased inflation pressure was 2.4% for frontal cases and 7.1% for oblique cases. For the frontal cases, reduced inflation pressure had the most influence (5%) at the most inferior locations (− 10 mm). For the oblique cases, the + 10 mm and + 30 mm locations had the greatest influence of 9.5% and 7.5%, respectively.

The secondary ball changed PAV by between + 21 and − 14% (Fig. 4.b.i to b.iv,). For every instance that the primary ball resulted in a smaller PAV at 1.0 bar it also resulted in a smaller PAV at 0.6 bar. This was consistent for instances where the primary ball resulted in larger PAV. All locations where the secondary ball increased PAV occurred at the most inferior height (− 10 mm) with a mean increase of 11.8%. The locations where the secondary ball decreased PAV all occurred at a height of + 10 mm or above with a mean decrease of 9%.

#### Sensitivity Analysis for PAA

Figure 5.a.i to b.iv) presents the results for PAA which ranged from 3.1 to 5.8 krad/s^2^. Across all impacts, reduced inflation pressure resulted in an average decrease in PAA of 11%. The magnitude of PAA was in the range 47–87% of the CST surmised from literature [18, 20, 21, 24, 25, 42].

For the frontal cases (Fig. 5.a.i, a.ii, b.i and b.ii) PAA ranged from 3.1 to 4.6 krad/s^2^. There was a local maximum across six of the discrete impacts in the neutral-frontal cases for both pressures. These were across the entire row at + 30 mm and extended into the two most lateral impacts of the + 10 mm row. The maximum PAA for the flexed-frontal impacts (Fig. 5.a.ii and b.ii) was 4.5 and 4.1 rad/s^2^ for the 1.0 and 0.6 bar conditions, respectively and these both occurred at + 30 mm height. The first column of the neutral-frontal cases and the flexed-frontal cases showed the same trend of local minima at the most superior and most inferior locations.

PAA ranged from 3.6 to 5.8 rad/s^2^ for the oblique impacts and was on average 25% greater than the equivalent frontal locations. For a given vertical offset row, the flexed-oblique impact was on average 3% smaller than the corresponding neutral-oblique impact. The 1.0 bar and 0.6 bar cases both exhibited increased PAA magnitude as the impact location became more superior. The PAA results for the oblique cases at the 1.0 bar condition peaked at 87% of the CST and the 0.6 bar conditions peaked at 77% of the threshold. Reduction in PAA due to decreased inflation pressure was on average 15% for the oblique impacts (Fig. 5.b.iii and b.iv) compared to 9.7% for the frontal impacts (Fig. 5.b.i and b.ii). Reduction in PAA was greatest across the most inferior locations (12.4% for the frontal cases and 20.5% for the oblique cases) and least across the most superior locations (7.4% for the frontal cases and 10.5 for the oblique cases).

In every case the secondary ball was smaller magnitude than the primary ball. The difference ranged from 6% to 51% smaller PAA. For the most inferior locations (− 10 – 10 mm) the 1.0 bar condition exhibited a difference between ball types that was between 4 and 17% larger than the 0.6 bar equivalent. At the most superior location (50 mm) the 0.6 bar condition exhibited the larger difference between ball types (4% larger).

#### Sensitivity Analysis for DAMAGE Metric

The DAMAGE predictor of brain DAMAGE ranged from 0.083 to 0.111 mm/mm and from 55 – 74% of the CST for DAMAGE (Fig. 6.a.i to b.iv). Across all impacts reduced inflation pressure resulted in an average decrease in DAMAGE of 1% (Fig. 6).

The neutral-frontal impacts at 1.0 bar (Fig. 6.a.i) ranged from 0.089 to 0.105 mm/mm and showed a local maximum across the most inferior row of impacts (− 10 mm). The 0.6 bar neutral-frontal condition (Fig. 6.b.i) ranged from 0.089 to 0.097 mm/mm and exhibited twin maxima across the most inferior row (same as 1.0 bar) and across the two most superior rows.

The flexed-frontal impacts (Fig. 6.a.ii and b.ii) were on average 7% less than the equivalent neutral-frontal impact. Both the flexed-oblique cases (0.6 and 1.0 bar) exhibited a local minimum at the 10 mm vertical location which was 3% (0.6 bar) and 9% (1.0 bar) less than the respective flexed-frontal maxima. The 1.0 bar condition had a distinct maximum at the most inferior location whilst the 0.6 bar condition exhibited approximately equal maxima both superior and inferior of the 10 mm vertical height. This followed the same trend as the neutral-frontal cases.

The largest peak values for DAMAGE occurred in the neutral-oblique cases (0.111 mm/mm at 1.0 bar and 0.104 mm/mm at 0.6 bar) which corresponded to 74% and 69% of the CST. The oblique impacts ranged from 0.094 to 0.111 mm/mm for the 1.0 bar condition and from 0.091 to 0.104 mm/mm for the 0.6 bar condition which made the smallest oblique magnitudes similar to the largest frontal magnitudes. The difference between the neutral-frontal impacts and the flexed-frontal impacts was minimal (≤3%). The oblique impacts exhibited greater magnitude as the impact became more inferior.

For the most inferior locations (− 10 mm) reduced inflation pressure resulted in an average decrease in DAMAGE of 7% with a small range across head orientations and neck flexion angles (3 to 9%). At 10 mm the frontal impacts averaged a decrease of 1% whilst the oblique impacts averaged a decrease of 8%. At 30 and 50 mm the frontal impacts averaged an increase in DAMAGE of 6 and 4% compared to the oblique impacts that averaged a decrease of 5 and 2%.

The primary ball was between − 13 and 19% different to the secondary ball. For the more inferior impacts (− 10 and 10 mm) the primary ball was lower magnitude than the secondary ball by an average of 9% including the two oblique configurations. For the more superior impacts (30 and 50 mm) the primary ball was greater magnitude than the secondary ball by an average of 7%. In every case where the secondary ball showed greater magnitude than the primary ball, the difference was larger at 1.0 bar than at 0.6 bar. When the primary ball was greater magnitude than the secondary ball the difference was larger at the 0.6 bar condition.

### Magnitude Relative to CST

**Fig. 7:**
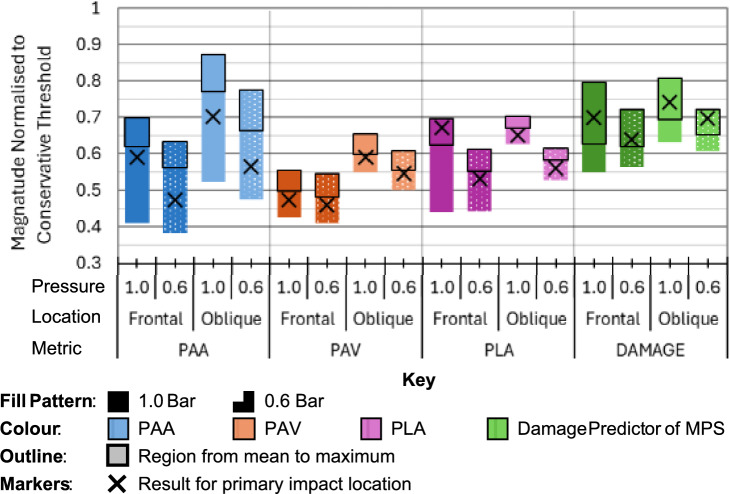
Box plots of normalised results against respective CST (PAA, PAV, PLA and DAMAGE) representing the range of results, mean of results and result for the primary impact grouped by pressure and impact type (oblique or frontal)

Figure 7 plots the magnitude of each metric normalised against each respective CST established in Table 3. Normalised magnitudes ranged from 0.38 to 0.87. In Fig. 7 the magnitude region from the mean to the maximum result for each case is highlighted which shows the region that corresponds to average or above magnitude. In five out of 16 cases the primary impact produced above average magnitude. Four occurrences were for the DAMAGE metric, and one was for the PLA frontal impact at 1.0 bar. In every case the maximum of the oblique impact was greater than or equal to the maximum for the frontal impact. For PAA and PAV the frontal cases were consistently between 0.17 (1.0 bar PAA) and 0.05 (0.6 bar PAV) smaller than the oblique cases. For PLA and DAMAGE, the maxima for frontal and oblique were equal in every case.

The overall normalised mean for both PAA and DAMAGE were equal largest at 0.65 and on average 35% below their respective thresholds (from Fig. 7 and Table 6). For PAA the overall mean (0.65) was 12% greater than the mean for the primary impacts (0.58). In contrast, for DAMAGE the overall mean (0.65) was 6% less than the mean for the primary impacts (0.69). The overall mean for PLA was 0.61 which was very similar to the mean for the primary impacts (0.60). This was biased by the frontal 1.0 bar result where the primary impact was 0.05 greater than the respective frontal 1.0 bar mean. For the other conditions, the PLA of the primary impact was on average 0.02 less than the respective means. The overall mean for PAV was 0.53 which was 0.01 greater than the mean for the primary impacts (0.52). PAA results had an average overall range of 0.3 which was the largest of the four metrics. The average range was 0.17 for DAMAGE, 0.15 for PLA and 0.12 for PAV. The mean magnitude reduction due to reduced inflation pressure was greatest for PAA and PLA (12%). PAV and DAMAGE both exhibited a more modest mean reduction of 5%. For PAA, PAV and PLA the percentage reduction in mean magnitude due to reduced inflation pressure was consistent with the percentage reduction in mean maximum magnitude.

**Table 6:** Summary of the net effect of decreased inflation pressure on head kinematics [n = 204]

	Effect of reduced inflation pressure on magnitude			
	Frequency			Mean magnitude change
	Decrease	No change*	Increase	
PLA	32	2	0	− 11%
PAV	27	6	1	− 4%
PAA	34	0	0	− 12%
DAMAGE	19	3	12	− 2%
All Metrics	112	11	13	− 7%

* < 1.5% change or statistically insignificant change

### Overall Effect of Reduced Inflation Pressure

A summary count of the frequency of occasions where reduced inflation pressure resulted in a magnitude change and the mean overall magnitude change is presented in Table 6. For PLA and PAA, reduced inflation pressure reduced the respective magnitude for every case except two unchanged instances. For PAV the magnitude was mostly reduced but in six out of 34 cases it was unchanged and in one case it increased. The DAMAGE metric decreased with decreased inflation pressure for more than half of cases. However, there were 12 instances where DAMAGE was increased by decreased inflation pressure. The mean effect of reduced inflation pressure was a reduction in magnitude across all four metrics.

## Discussion

### The Influence of Inflation Pressure on Ball Characteristics

The characterisation of the two ball types showed that inflation pressure was the dominant factor for COR and stiffness which is consistent with findings from literature [43]. This is important because these characteristics are most likely to affect ball-head interaction. COR was reduced by an average of 3.8% with reduced inflation pressure and this dominated the overall range in COR (6.1%). The ball type and its orientation were found to have a lesser influence over COR than inflation pressure. Similarly, the mean stiffness difference between the two ball types was minor (3%) compared to the 33% mean difference due to reduced inflation pressure. Both balls conform to the FIFA Quality Pro Standard [33] which partially explains why the difference between them across all characteristics (including mass, MOI and diameter) was small. That said, compressive stiffness was the characteristic that differed the most between the two balls. The percentage change in stiffness when going from 0.6 bar to 1.0 bar was 36% and 29% for the primary and secondary balls, respectively. The secondary ball was the stiffer ball at 0.6 bar whereas the primary was the stiffer at 1.0 bar. This difference is explained by their individual constructions, indicating that a greater proportion of the Al Rahila’s stiffness was from the shell construction rather than the inflation pressure.

### The Influence of Inflation Pressure on Ball-Head Impact Response

Reduced inflation pressure resulted in lower magnitudes for 82% of conditions across the four kinematic metrics, suggesting that this was an overall positive intervention. The reduction to 0.6 bar inflation pressure resulted in an overall mean reduction in PLA of 12% and a maximal reduction of 21%. Pereira et al. [44] reported that reduced inflation pressure resulted in a 6% reduction in PAV and a 12% reduction in PLA which agrees with the findings of this study. Cecchi et al. [35] found that a 0.14 bar reduction in inflation pressure reduced PLA by 8%. Scaling to match the magnitude change in inflation pressure in this study would give a predicted reduction of 21%. The maximum reduction in this study (21%) was in the primary frontal location, which was closest to the impact location of Cecchi et al. [35]. This comparison highlights the sensitivity of head kinematic results to impact location and the potential to overestimate the effectiveness of reduced inflation pressure and underestimate maximal PLA. Reduced inflation pressure also resulted in increased and unchanged magnitudes for the remaining 10% and 8% of results, despite this the overall maxima within each metric always decreased with reduced inflation pressure. This was also true within each orientation/flexion configuration except for PAV in the neutral-frontal configuration where the maxima were essentially unchanged (Fig. 4.b.i). Nonetheless the maxima for PAV in frontal configurations (Fig. 4.b.i and b.ii) were substantially smaller than the maxima of the oblique configurations (Fig. 4.b.iii and b.iv) diluting the significance of this observation. These findings suggest that reduced inflation pressure is not a unilateral solution, even though the reductions were generally larger magnitude and more numerous than the increases. The kinematics reported in this study were found to be in good agreement with those in the literature (Table 7). For studies using ATDs the filtering method is specified in Table 7 to provide a fairer comparison to the results in this study. The filters for PLA and PAA calculation used in this study were SAE J211 compliant (PAA calculation used a prefilter of 300 Hz on raw angular velocity before differentiation). The PAV filter was set at 1650 Hz as opposed to the SAE J211 compliant 300 Hz. A sensitivity analysis showed that this resulted in a mean increase of 0.89% to the PAV results by retaining slightly more of the high frequency content caused by the relatively short contact time.

**Table 7:** Summary of comparable literature to the results from this study

Reference	Description			PLA (g)	PAV (rad/s)	PAA (krad/s^2^)	MPS ^Δ^ (mm/mm)
This Study	17 m/s	Frontal at 1.0 bar		31.8	11.3	4.1	0.09 95th
		Frontal at 0.6 bar		28.2	11.0	3.7	0.09 95th
		Oblique at 1.0 bar		34.2	13.6	5.1	0.10 95th
		Oblique at 0.6 bar		29.7	12.6	4.4	0.10 95th
[46]	35 m/s at 0.9 bar FEA *(supplement data)*		Frontal	62.1		3.8	< 0.01 undefined
			Side	95.3		8.2	0.04 undefined
[35]	Frontal at 17.3 m/s HIII Accelerometer and ARS with SAE J211 filtering		0.55 bar	29.8 ^#^		1.3^#^	
			0.62 bar	30.9 ^#^		1.4^#^	
			0.76 bar	33.5 ^#^		1.6^#^	
[16]	Frontal at 16 m/s and 0.8 bar In vivo and FEA			31.9	9.1	2.1	0.11 95th^#^
[47]	Frontal at 9.0 m/s In vivo		0.4 bar	4.8		0.59	
			0.54 bar	5.4		0.62	
[34]	17 m/s at 0.69 bar HIII and FEA Nine accelerometer pack with CFC1000 filter		Frontal	26.2		3.0	0.17 95th
			Oblique Boss	30.2		4.2	024 95th
[26]	11.2 m/s at 0.83 bar In vivo and FEA		Frontal	17.5	5.5	1.1	0.04 95th^#^
			Side	12.3	10.2	1.4	0.05 95th^#^
[44]	8.3 m/s ‘Rotational headers’ In vivo		1.03 bar	14.4	16.2		
			0.59 bar	12.8	15.3		

^#^- digitised from graphs

^Δ^MPS or MPS predictor with percentile criteria in subscript

This study used a nominal impact velocity of 17 m/s to represent the most common heading scenario of a corner kick [45]. Perkins et al. [46] conducted impacts at 35 m/s, which is close to maximal velocity for a shot or goal kick. Scaling their results by velocity would result in PLA and PAA of approximately 30 g and 1.8 krad/s^2^ and 46 g and 4.0 krad/s^2^ for frontal and side impacts, respectively, which is in good agreement with the results presented in this study. The magnitude for PLA agreed well with the results of Cecchi et al. [35] even though the magnitude for PAA (1.3 – 1.6 krad/s^2^) was lower than this study. This is partially explained by the frontal impact location. The closest equivalent from this study (neutral-frontal) was in the range of 2.6–3.1 krad/s^2^ which is closer in agreement than the average results of this study. Barnes-Wood et al. [16] found PAV and PAA to be smaller in magnitude than this study. This is partially explained by their frontal only impact location choice, which compared more closely with this study’s primary frontal impact and resulted in PAV of 9.3 rad/s at 0.6 bar. It could also be explained by the in vivo nature of the study and the associated instrumentation challenges such as low sampling rate (1 kHz) or un-validated adhesion between the dentition mounted iMG and the wearer. Ferdousi et al. [34] tested at 0.69 bar and their results agreed well with the range of results from this study at 0.6 bar despite using the alternative instrumentation method (nine accelerometer package) to calculate PAV and PAA.

The results of this study found that the oblique impacts generated higher angular kinematics than the frontal impacts and that whilst reduced inflation pressure did reduce angular kinematics, PLA was reduced to a greater extent. Ferdousi et al. [34] also found that the oblique impact generated greater PLA and PAA. Similarly, Perkins, Bakhtiarydavijani, & Prabhu [48] found their simulation of a side impact to generate more angular acceleration and reduced inflation pressure to be more influential on Head injury Criterion [49] (which is PLA derived) than Brain Injury Criterion [50] (which is PAV derived). Huber et al. [26] tested at significantly slower impact velocity (11.2 m/s) than this study and when scaled to represent the 17 m/s impact velocity, their results contrast with the results of this study and Ferdousi et al. [34], suggesting that the side impact had lower PLA than the frontal impact. This is difficult to explain without a more specific definition of the impact location. Preira et al. [44] tested at almost the exact same pressures as this study. Their impact location was not well quantified which makes the magnitude values difficult to explain. When scaled for impact velocity their PLA values agree well with this study (29.5 and 26.2 for 1.03 and 0.59 bar, respectively). However, their scaled PAV results are much greater than those in any other study (33.2 and 31.3 for 1.03 and 0.59 bar, respectively). Their study involves human participants actively directing the ball with a rotational heading motion which could explain the high PAV magnitudes. Voluntary active head rotation has been shown to generate between 12.6 and 25.0 rad/s PAV [51]. The influence of this active motion on resulting head kinematics needs to be considered further when reconstructing headers in the laboratory and simulating computationally. Furthermore, their study used an inertial measuring unit secured by a head band and positioned over the occiput. This approach has been shown to result in a less coupled sensor-skull response and an overestimation of angular kinematics [52].

The predicted MPS from the calculated DAMAGE metric in this study and that presented by Barnes-Wood et al. [16] were in good agreement. The results of Huber et al. [26] can be scaled by velocity difference to give an estimated MPS of 0.06 and 0.08 which showed good agreement with the minimum values in this study. In contrast, the results of Ferdousi et al. [34] suggest that the results of this study were low. It is interesting to note the Ferdousi et al. [34] use the UCDBTMv2.0 FEA model whilst Huber et al. [26] used the KTH FEA model. The MPS results from the aforementioned studies were all in reasonable agreement with each other whilst the results of Perkins et al. [46] were significantly smaller, especially considering their high impact velocity. It is possible that they were reporting a lower percentile MPS (e.g., 90^th^ percentile).

The sensitivity analysis of this study included a maximum offset of 60 mm (vertical and horizontal) from the primary frontal and oblique impact locations. Based on previous (unpublished results), this is within the accuracy of other commonly used ball launch devices and represents a reasonable deviation in achievable location. The ranges of results within this sensitivity analysis were 56, 35, 37 and 32% (at 1.0 bar) and 51, 32, 28 and 22% (at 0.6 bar) for PAA, PAV, PLA and DAMAGE, respectively. This represents the possible uncertainty ranges for studies with typical control over impact location and studies that try to represent heading by a single impact location/configuration. Studies with low control of impact velocity would further increase measurement uncertainty. The results of this study suggested that one or two impacts were insufficient to represent the range of impacts in football heading. This was determined by the excellent impact accuracy in this study. The primary impacts were on average − 7, − 2, 0 and + 5% different to the overall mean and − 16, − 7, − 5 and − 7% less than the maxima (PAA, PAV, PLA and DAMAGE, respectively). The results of this study suggest that at least four permutations of impact location and orientation were required to fully represent the range from average to severe impacts when considering the four metrics presented in Figure 3, 4, 5 and 6. In most research scenarios it is excessive to represent both average and severe cases. A graph summarising the results of different metrics (supplementary data) identified that a neutral-oblique impact in the range + 40 to + 50 mm vertical offset could on balance reflect the overall worst case. However, frontal and oblique impacts created fundamentally different kinematics and both should be represented with a respective worst case. The results of this study suggests also testing a neutral-frontal impact in the region 0 to + 30 mm vertical and + 40 to + 60 mm horizontal offsets.

All tests were conducted with a Hybrid III head constrained by a Hybrid III neck due to their wide adoption in other studies, enabling fair comparison with reported data [34, 35]. Previous studies have not typically reported the orientation of the neck relative to the lower mounting bracket (i.e., initial flexion angle) and so this study investigated its influence on the resulting head kinematics. In every instance, an increased neck flexion angle either reduced the metric magnitude or had no discernible effect. These findings indicate the importance of reporting the tested orientation and support the idea that taught heading technique may influence the head impact event experienced by a player in the real world [53, 54]. It is also reported that ball to head impacts with the most severe outcomes have been observed from unexpected/unsighted impacts [9], where the neck is likely to be unbraced. The overly stiff nature and simplicity of the Hybrid III neck not only reduce the level of variability in its response but also reduce the degree to which it reasonably represents the typical neck stiffness of the player heading the ball. The Hybrid III neck is known to be overly stiff [55–57] and most closely represents a highly braced passive (i.e., not moving) neck. It does not represent an unbraced player who did not anticipate the impact or an actively braced player who is intentionally directing the ball through head motion. The unbraced (low stiffness) and higher ball impact velocity (e.g., clearance kick) have been related to increased risk of symptomatic outcome [46]. Furthermore, the Hybrid III neck is designed for frontal impacts and its response is only validated for sagittal plane motion due to its limited ability to axially rotate and its overly stiff response to lateral flexion. Recent developments in surrogate necks have focused on representing ranges of motion and stiffnesses in each anatomical plane that are tuneable from unbraced to braced responses [58]. Future research should also focus on the influence of the chosen surrogate devices for representing real world impacts in the laboratory. The two neck orientations showed fundamentally different impact kinematics, indicating the value of exploring the spectrum of neck axial rotation angles in future research. Because the HIII neck was excessively stiff in axial rotation there would have been limited value of investigating the full spectrum of axial rotation dominated impacts without a more biofidelic neck.

Ball type was found to be a significant factor influencing head kinematics with a mean magnitude difference between the balls of 9%, 10%, 22% and 9% for PLA, PAV, PAA and DAMAGE, respectively. For PAA, the primary ball was always larger magnitude than the secondary ball and the magnitude difference became less as the impact became more superior. For PAA the magnitude difference was always greater at 1.0 bar than 0.6 bar correlating to the change in stiffness but the secondary ball was also always greater magnitude at 0.6 bar where it was the less stiff ball. In contrast for PAV and DAMAGE the secondary ball expressed larger magnitudes at the most inferior impacts and the primary ball showed greater magnitudes for the more superior impacts. The largest differences between the balls were always at the most extreme impact locations (flexed-frontal − 10 mm, neutral-frontal − 10 mm and neutral-frontal + 50 mm). Ball characterisation showed that mass, diameter and MOI of the two balls were similar as would be expected for two balls conforming to the FIFA Quality Pro Standard [33]. Greater magnitude differences were observed for compressive stiffness and COR, and the balls had different surface finishes by design. There was a high frequency of statistically significant differences in heading kinematics between the ball types which indicates that further work should focus on characterising the compressive stiffness, COR and surface finish to understand how these properties affect kinematic differences for heading.

No single impact in this study exceeded the already conservative CST values established in Table 3. The average magnitude across all metrics and all impacts was 61% of the respective CSTs and the average of the maximum for each metric was 69%, therefore agreeing with the low frequency of symptomatic outcomes from ball to head contacts observed in the game [7, 8]. Based upon maximal values and mean values the metrics PAA (for oblique impacts) and DAMAGE (for all impacts) were closest to their respective thresholds. At 1.0 bar DAMAGE peaked at 80% threshold for both the frontal and oblique impacts and the metric PAA included the single largest result (87%) for one of the oblique impacts.

The results of this study concurred with literature that reduced inflation pressure was a positive mitigation strategy towards reducing head impact kinematic magnitudes in heading but employed significantly more rigour towards understanding the interaction effects of ball type, impact location, and head orientation on four different metrics. A statistically significant reduction in magnitude was the modal finding (82%), however there was a small subset of results where reduced inflation pressure did not change kinematic magnitude (8%) and another where it paradoxically increased magnitude (10%). These findings highlight the benefit of the rigorous sensitivity analysis in this study and show that this level of thoroughness was required to form a complete investigation. Nonetheless they formed the minority, being both smaller in magnitude and less frequent than the decreases in magnitude. This led to the overall conclusion that on balance the net effect of reduced inflation pressure was a positive intervention towards reducing the magnitude of head kinematics in heading.

## Supplementary Information

Below is the link to the electronic supplementary material.

Supplementary file1 (DOCX 198 kb)
