# Supplementary material for: Does Reduced Ball Inflation Pressure in Association Football Decrease Head Impact Kinematics?
Source: Ann Biomed Eng. 2025 Aug 4;53(11):3109–25. doi: 10.1007/s10439-025-03804-0 (PMC12575582; doi:10.1007/s10439-025-03804-0)
Supplement: Supplementary file 1 — Supplementary file1 (DOCX 198 kb) [file 10439_2025_3804_MOESM1_ESM.docx]

# Supplementary Data


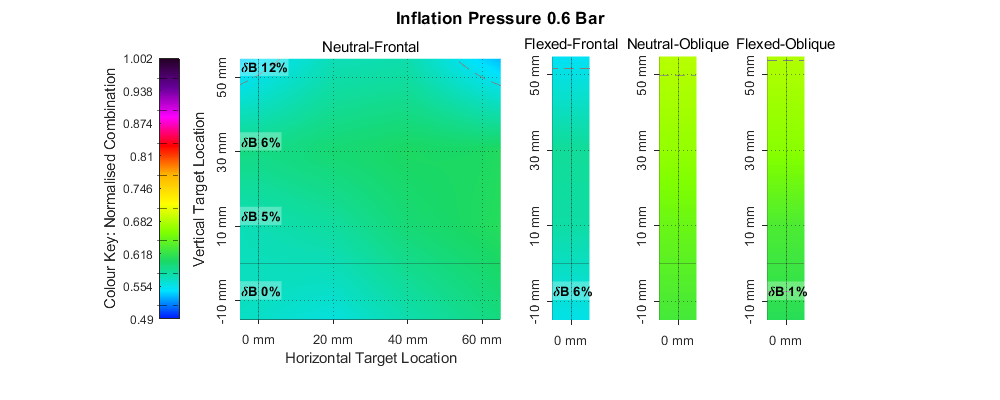


Figure 8 – Heat maps showing the consolidated magnitude across all metrics at both pressures normalised against the respective CST for each metric with the local high spots for the frontal and oblique impacts highlighted in the pink circle (Supplementary Data).
